# Long-Term Treatment with Liraglutide, a Glucagon-Like Peptide-1 (GLP-1) Receptor Agonist, Has No Effect on β-Amyloid Plaque Load in Two Transgenic APP/PS1 Mouse Models of Alzheimer’s Disease

**DOI:** 10.1371/journal.pone.0158205

**Published:** 2016-07-15

**Authors:** Henrik H. Hansen, Katrine Fabricius, Pernille Barkholt, Pernille Kongsbak-Wismann, Chantal Schlumberger, Jacob Jelsing, Dick Terwel, Annelies Termont, Charles Pyke, Lotte Bjerre Knudsen, Niels Vrang

**Affiliations:** 1 Gubra, Agern Allé 1, DK-2970 Hoersholm, Denmark; 2 reMYND NV, Gaston Greenslaan 1, B-3001 Leuven-Heverlee, Belgium; 3 Diabetes Research, Novo Nordisk A/S, Novo Nordisk Park, DK-2760 Maaloev, Denmark; University of S. Florida College of Medicine, UNITED STATES

## Abstract

One of the major histopathological hallmarks of Alzheimer’s disease (AD) is cerebral deposits of extracellular β-amyloid peptides. Preclinical studies have pointed to glucagon-like peptide 1 (GLP-1) receptors as a potential novel target in the treatment of AD. GLP-1 receptor agonists, including exendin-4 and liraglutide, have been shown to promote plaque-lowering and mnemonic effects of in a number of experimental models of AD. Transgenic mouse models carrying genetic mutations of amyloid protein precursor (APP) and presenilin-1 (PS1) are commonly used to assess the pharmacodynamics of potential amyloidosis-lowering and pro-cognitive compounds. In this study, effects of long-term liraglutide treatment were therefore determined in two double APP/PS1 transgenic mouse models of Alzheimer’s disease carrying different clinical APP/PS1 mutations, *i*.*e*. the ‘London’ (hAPP_Lon/_PS1_A246E_) and ‘Swedish’ mutation variant (hAPP_Swe_/PS1_ΔE9_) of APP, with co-expression of distinct PS1 variants. Liraglutide was administered in 5 month-old hAPP_Lon/_PS1_A246E_ mice for 3 months (100 or 500 ng/kg/day, s.c.), or 7 month-old hAPP_Swe_/PS1_ΔE9_ mice for 5 months (500 ng/kg/day, s.c.). In both models, regional plaque load was quantified throughout the brain using stereological methods. Vehicle-dosed hAPP_Swe_/PS1_ΔE9_ mice exhibited considerably higher cerebral plaque load than hAPP_Lon/_PS1_A246E_ control mice. Compared to vehicle-dosed transgenic controls, liraglutide treatment had no effect on the plaque levels in hAPP_Lon_/PS1_A246E_ and hAPP_Swe_/PS1_ΔE9_ mice. In conclusion, long-term liraglutide treatment exhibited no effect on cerebral plaque load in two transgenic mouse models of low- and high-grade amyloidosis, which suggests differential sensitivity to long-term liraglutide treatment in various transgenic mouse models mimicking distinct pathological hallmarks of AD.

## Introduction

Glucagon-like peptide 1 (GLP-1) receptor agonists have prominent insulin-dependent glycemic effects and are currently in use for the treatment of type 2 diabetes [1]. As peripherally administered GLP-1 receptor agonists can access the brain [2–4] and GLP-1 receptors are widely expressed in the CNS [5–7], this has led to an increasing appreciation that GLP-1 receptor agonists may also have important central effects beyond glucose homeostasis and weight control. As a consequence of the discovery of neurotrophic, neuroprotective, neurogenesis and memory-enhancing effects induced by enhanced GLP-1 receptor function [8–10], considerable efforts have in the last decade been made to assess possible disease-modifying properties of GLP-1 receptor agonist treatment in neurodegenerative diseases [11,12]. Notably, GLP-1 receptor agonists have shown neuroprotective properties in various preclinical models of Alzheimer’s disease (AD), in particular in the context of β-amyloid aggregation and accumulation, which represent a major histopathological hallmark of AD [13]. Native GLP-1 reduces β-amyloid oligomer levels and toxicity in cultured primary neurons and neuronal-like cell lines [10,14,15], and similar effects have been reported with exendin-4, a protease-resistant GLP-1 analogue [16–18]. Importantly, the findings of amyloidosis-reducing effects of pharmacologically enhanced GLP-1 receptor function have been extended to *in vivo* experimental AD models. Accordingly, the GLP-1 receptor agonists liraglutide and lixisenatide have been demonstrated to ameliorate memory impairment and improve hippocampal synaptic plasticity in rats exposed to an intrahippocampal injection of β-amyloid peptides [19,20]. In support of reduced vulnerability to amyloidosis-associated neurotoxicity, subchronic (3 weeks) exendin-4 treatment has been shown to reduce soluble β-amyloid levels, to lower cortical amyloid plaque load and improve memory performance in a double transgenic APP/PS1 mouse model of amyloidosis [21]. In this transgenic model, liraglutide and lixisenatide have also been demonstrated to evoke similar effects on memory function and plaque burden [22,23], as well as improving markers of synaptic plasticity and to stimulate neuroproliferative activity [23–25].

Double transgenic APP/PS1 mouse models carry genetic mutations of human APP (amyloid protein precursor) and PS1 (presenilin-1), being recognized as high-risk factors for development of early-onset familial dementia [26]. Therefore, APP/PS1 mouse models are commonly used to assess the pharmacodynamics of potential pro-cognitive and amyloidosis-lowering compounds [27,28]. Due to the many mutation variants of the APP and PS1 genes [29], several double transgenic APP/PS1 mouse model lines have been generated for studying the histopathological and neurobehavioral impact of clinical relevant mutations in the APP and PS1 genes linked to AD [30–32]. It is important to recognize that transgenic APP/PS1 mouse lines display differences in regard to the onset, brain regional distribution and severity of plaque accumulation, as well as showing age-dependent and distinct neurobehavioral phenotypes [27,28,33]. Hence, the choice of transgenic APP/PS1 mouse line may potentially influence the efficacy profile of test compounds, making it important to study effects of GLP-1 receptor agonist treatment across different transgenic APP/PS1 mouse strains.

We have recently reported that liraglutide showed neuroprotective effects in two mouse models of AD devoid of β-amyloid plaques, i.e. non-transgenic senescence-accelerated mouse prone 8 (SAMP8) mice [34] and hTauP301 transgenic tauopathy mice [35]. To examine the effect of liraglutide administration in the specific context of β-amyloid plaque pathology, the aim of the present study was therefore to assess neurobehavioral and molecular effects of long-term liraglutide treatment in two APP/PS1 transgenic mouse models of Alzheimer’s disease carrying different clinical APP/PS1 mutations. To this end, we characterized transgenic mouse models representing the ‘London’ (hAPP_Lon/_PS1_A246E_) and ‘Swedish’ mutation variant (hAPP_Swe_/PS1_ΔE9_) of human APP, respectively [30,36]. The two transgenic double APP/PS1 transgenic models display distinct age-dependent progression and severity of amyloid plaque burden [30,36–40], allowing for the assessment of effects of liraglutide administration in the context of differential APP/PS1 mutation-induced β-amyloid overexpression and associated cognitive deficits. To account for differential progression rates of amyloidosis in the two AD models, liraglutide treatment was initiated in both models at individual ages when only very low-grade amyloidosis would be expected.

## Material and Methods

### Animals

All animal experiments were conducted in accordance with internationally accepted principles for the care and use of laboratory animals, and were approved by the Danish Council for Animal Research (study-specific licence 2008/561-1565).

#### Transgenic hAPP_Lon_/PS1_A246E_ mice

5-month old females (n = 54) of a double transgenic mouse model of Alzheimer’s disease in FVB/N x C57Bl/6J (F1) background (FVB/N) were employed, expressing both the human clinical ‘London’ mutant V717I variant of the amyloid precursor protein (hAPP_Lon_) and a clinical mutant variant of human presenilin 1 (hPS1_A246E_) under control of the neuron-specific murine thymus-1 (*thy1*) gene promoter. Wild-type mice (n = 17) with similar genetic background were used as non-transgenic controls. The F1-hybrid strain was a crossing of heterozygous hAPP_V717I_ males in C57Bl/6J background with wild-type FVB/N females, and double transgenic mice overexpressing hAPP_Lon_ and hPS1_A246E_ were generated by cross-breeding the single hAPP_Lon_ mutant with homozygous hPS1_A246E_ mice [30]. The age-dependent behavioural and histopathological phenotype of double transgenic hAPP_Lon_/hPS1_A246E_ mice has been reported previously [30,37,41]. All hAPP_Lon_/hPS1_A246E_ mice were genotyped by two independent PCR assays with primers specific for the mutant hAPP and hPS1 sequence on DNA extracts from tail biopsies sampled at the age of three weeks and three weeks before treatment start, respectively. Age-matching and treatment randomization was thereafter performed, the mice were re-caged (n = 6 per cage) and allowed to habituate to the new cage context for two weeks. Cage-specific grouping was thereafter kept identical throughout the study. All mice were identified by ear marks and tail-marked with waterproof ink every week. During the study, the mice were housed in individually ventilated (IVC) macrolon T2 cages equipped with solid floors and a layer of bedding. Environmental parameters were as follows: temperature 24 ± 2°C; relative humidity: 55 ± 5%; air changes 75–80 times per hour; lights off 6 am-6 pm. Mice had ad libitum access to standard mouse chow (Ssniff® Ms-H, Ssniff Spezialdiäten GmbH, Soest, Germany) and pre-filtered, sterile water. The amounts of food and water were checked on a daily basis, supplied when necessary and refreshed once weekly.

#### Transgenic hAPP_Swe_/hPS1_ΔE9_ mice

7-month old females (n = 42) of a double transgenic mouse model of Alzheimer’s disease in C57Bl/6J background were employed, expressing both the human clinical ‘Swedish’ mutant K595N/M596L variant of the amyloid precursor protein (hAPP_Swe_) and an exon-9-deleted clinical mutant variant of human presenilin 1 (hPS1_ΔE9_) under control of the neuron-specific murine prion protein gene promoter [32]. Female mice were used, as female APP/PS1 mice typically bear a heavier β-amyloid burden and higher plaque number compared to male mice of the same age [27,42]. The transgenic hemizygous hAPP_Swe_/hPS1_ΔE9_ mice (strain name: B6.Cg-Tg(APPswe,PSEN1dE9)85Dbo/Mmjax, stock number 034832-JAX, https://www.mmrrc.org/catalog/sds.php?mmrrc_id=34832), 5–8 weeks of age at arrival, and female wild-type littermate controls (n = 20) were obtained from The Jackson Laboratory (Bar Harbor, ME). Upon arrival to the animal facility, the mice were equipped with a unique microchip (Pet ID Microchip, E-vet, Haderslev, Denmark), implanted under light CO_2_ anaesthesia. Animals were thereafter identified using microchip scanner (WS-1 weighing station, MBrose ApS, Denmark) connected to a laptop software (HM02Lab, Ellegaard Systems, Denmark). During the study, the mice were group-housed in polypropylene cages (n = 4–5 per cage) with stainless steel mesh lids mounted with feeders containing regular chow (Altromin 125, Brogaarden, Denmark). Cage-specific grouping was kept identical throughout the study. Mice were housed in ventilated cabinets (Scanbur Technology A/S, Denmark). Cages were cleaned once weekly but not before any behavioural studies. Environmental parameters were as follows: temperature 22 ± 1°C; relative humidity: 50 ± 10%; air changes 15 times per hour; lights on-off 6am:6pm. The amounts of food and water were checked on a daily basis, supplied when necessary and refreshed twice weekly.

### Drug treatment

#### Transgenic hAPP_Lon_/hPS1_A246E_ mice

Drug treatment was initiated when the animals were 5-month old, i.e. before onset of amyloidosis in hAPP_Lon_/hPS1_A246E_ mice starting at 6 months of age [30,37,38]. Two weeks prior to treatment start, transgenic mice were randomized into either vehicle dosing (0.1% bovine serum albumin (BSA) in PBS, n = 18) or liraglutide treatment (100 μg/kg/day, n = 18; 500 μg/kg/day; n = 18) according to body weight. Liraglutide (molecular weight, 3751 g/mol) was from Novo Nordisk A/S (Maaloev, Denmark). Age-matched female wild-type littermate controls served as non-transgenic controls and were dosed with vehicle (n = 17). The mice were dosed subcutaneously (s.c.) once daily (1 ml/kg) for a total duration of 3 months. A dose-escalation scheme was implemented to reduce anticipated initial side-effects of liraglutide, including taste aversion and pica behavior, as GLP-1 receptor induced discomfort in rodents is transient and typically only observed within the first 2–3 days of dosing [43]. Dosing was started with an initial dose of 25 μg/kg/day, and increased through daily increments (50-100-150-200-250-300-500 μg/kg/day) until reaching the target dose on treatment day 2 (100 μg/kg/day, equivalent to 26.6 nmol/kg/day, MW 3751) or day 7 (500 μg/kg/day, equivalent to 133 nmol/kg/day).

#### Transgenic hAPP_Swe_/hPS1_ΔE9_ mice

Drug treatment was initiated when the animals were 7-months old, corresponding to the age where low-grade amyloidosis is present in hAPPSwe/hPS1ΔE9 mice [36,44–46]. Three days prior to treatment start, transgenic mice were randomized into either vehicle dosing (0.1% bovine serum albumin (BSA) in PBS, n = 20) or liraglutide treatment (500 μg/kg/day; n = 22) according to body weight as well as mean baseline performance in a novel object recognition and T-maze task, respectively (see below). Age-matched female wild-type littermate controls served as non-transgenic controls and were dosed with vehicle (n = 20). The mice were dosed subcutaneously (s.c.) once daily (1 ml/kg) for a total duration of 5 months. Step-wise dose increments were implemented similar to that indicated above until reaching the target dose on treatment day 7 (500 μg/kg/day).

In both studies, body weight was measured once daily during the entire dosing period.

### Drug exposure

Blood samples were taken from liraglutide-treated transgenic hAPP_Lon_/hPS1_A246E_ and hAPP_Swe_/hPS1_ΔE9_ mice to assess plasma drug exposure during the dosing period. Blood samples were obtained in mice before dosing and 4 hours post-dosing approximately 1½ month (hAPP_Lon_/hPS1_A246E_ mice) and 1, 3 and 5 months (hAPP_Swe_/hPS1_ΔE9_ mice) after treatment start, respectively. For blood collections, mice were anesthetized with isoflurane and approximately 100 μl of blood was obtained from the orbital plexus (hAPP_Lon_/hPS1_A246E_ mice) or tail vein (hAPP_Swe_/hPS1_ΔE9_ mice) with a 25 μl capillary glass tube in an EDTA-coated Eppendorf tube. Plasma was separated by centrifugation at 2,000 x g for 15 minutes at 4°C, plasma was collected and flash-frozen in liquid nitrogen before storage at -80°C until further analysis.

### Behavioral analyses in transgenic hAPP_Lon_/hPS1_A246E_ mice

#### Morris water maze task

The Morris water maze test (MWM) was applied to hAPP_Lon_/hPS1_A246E_ mice and corresponding wild-type controls after a total of 3 months of treatment. Mice were brought into the test room at least 30 minutes before the onset of training. They were kept in the south east corner behind a screen. A computer was in the south-west corner also behind a screen. The pool (a white, circular vessel ø 1.6 m) contained preheated water of 25 ± 1°C with titanium-dioxide as an odourless, nontoxic additive to hide the escape platform (0.5 cm beneath the water level). The platform (ø 16 cm) was located in the north-east quadrant. Around the pool, on screens and walls, a total of 9 cues were positioned. The cues were different in size and pattern, coloured black or black and white. Cues were visible from almost any position for mice in the pool, except when very close to the edge (the pool was filled with water up to 10 cm from the edge). Swimming of each mouse was video-taped and analyzed (Ethovision, Noldus information Technology, Wageningen, the Netherlands). For place navigation tests, mice were trained to locate the hidden platform in four blocks of four trials over four consecutive days. Each trial consisted of a forced swim test of maximum 90 seconds. Inter-trial interval was 60–120 min. The search path of each mouse to locate the platform in the four consecutive trials was measured, resulting in a learning curve. Mice were placed in the water bath in a pseudo-randomized order. For spatial reference memory evaluation, each animal was subjected to a probe trial at day 5. The platform was removed and each mouse was allowed to search for 60 seconds. Percentage time spent in target quadrant during the probe trial was measured as the key endpoint of the test. Statistical analysis was performed using Student’s T-test or ANOVA followed by Tukey’s or Bonferroni’s post hoc analysis where applicable. Comparisons were made between wild-type controls and hAPP_Lon_/hPS1_A246E_ mice on vehicle (genotype) and between hAPP_Lon_/hPS1_A246E_ mice on either vehicle or liraglutide administration (treatment).

### Behavioural analyses in transgenic hAPP_Swe_/hPS1_ΔE9_ mice

#### Novel object recognition task

The novel object recognition (NOR) task was applied to hAPP_Swe_/hPS1_ΔE9_ mice and corresponding C57Bl6 wild-type controls before (baseline evaluation performed three weeks prior to treatment start) and after a total of 5 months of treatment. The mice were habituated to an empty apparatus, a 42 × 27 × 21-cm (l x w x h) white plastic box for 5 minutes a day for 3 consecutive days prior to entry of objects. All animals were placed in the test room for a minimum of 1 hour on the first day of habituation and remained in the room behind a curtain for the reminder of the test-period. On the day of task acquisition (day 4), the mouse was placed in the testing apparatus with two identical objects for a total of 10 minutes. A minimum of 20 seconds of total exploration time of both objects was used as an inclusion criterion. 24 hours later the animal was reintroduced to the arena for 3 minutes without objects. Then, one of the original, familar objects was removed and a novel object added. Placement of the novel object alternated between right and left side (if side-preference occurred in the acquisition task, the novel object was placed in the opposite side). The mouse was allowed to explore both objects for 10 minutes, and the time each mouse spent sniffing or touching the new object was recorded (nose tip distance to object <2 cm). After each test, the arena was cleaned with a solution containing 10% ethanol to saturate the arena with odour. Total time spent exploring each of the two objects was recorded. The object discrimination index was defined as the amount of time exploring the novel object over the total time spent exploring both objects, and was used to measure object recognition memory [34].

#### Active avoidance T-maze task

The T-maze task was applied to hAPP_Swe_/hPS1_ΔE9_ mice and corresponding C57Bl6 wild-type controls before and after a total of 5 months of treatment. The T-maze task was introduced the week after completion of the baseline and final NOR task. The T-maze consisted of a black plastic alley with a start box at one end and two goal boxes at the other. The start box was separated from the alley by a plastic guillotine door that prevented movement down the alley until raised at the onset of training. An electrifiable floor of stainless steel rods ran throughout the maze to deliver a foot-shock using a scrambled grid floor shocker (Model E13-08, Coulbourn Instruments, Whitehall, PA). Mice were not permitted to explore the maze prior to training. A block of training trials began when a mouse was placed in the start box. The guillotine door was raised and a cue buzzer sounded simultaneously (doorbell type, at 50 dB); 5 seconds later a mild aversive foot-shock was applied with an intensity of 0.35 mA. The arm of the maze entered on the first trial was designated “incorrect” and the mild foot-shock was continued until the mouse entered the other goal box, which in all subsequent trials was designated “correct” for the particular mouse. At the end of each trial, the mouse was returned to its home cage until the next trial. Mice were trained until they made one active avoidance. The inter-trial interval was 30–40 sec. The number of trials to make one active avoidance was the measure of acquisition. Memory retention was tested one week later by continuing training until the mice achieved the criterion of making five active avoidances in six consecutive trials. The number of trials needed to reach this criterion was the measure of retention [34,47,48].

### Tissue sampling

Brains were collected from all mice the day after the last behavioural test. No dosing was performed on the termination day. The mouse was decapitated, the brain rapidly collected and post-fixed overnight in 4% paraformaldehyde (PFA). 4% PFA was replaced by PBS, 0.1% sodium azide after 24 hours and refreshed after 48 hours to remove last traces of PFA. Brains were stored at 4°C until processing.

### Assessment of β-amyloid plaque load and brain volume

The brain was divided into right and left hemisphere. One hemisphere from each animal, alternating systematically between left and right, was embedded overnight in paraffin blocks. Following embedding, the brains were cut exhaustively into 5 μm thick sections throughout the entire brain on a microtome (Microm HM340E, ThermoScientific) and sampled using systematic uniform random sampling principles with a sampling fraction of 50. Two series were collected and mounted on Flex IHC slides (DAKO, Denmark) and left to dry at 37°C for at least 24 hours before staining. In the event of poor technical quality, e.g. the presence of folds or breaks, the subsequent section was sampled instead. Sections were stained on a DAKO automated autostainer system (Link 48, DAKO, Denmark) according to the following protocol: paraffin-embedded sections were deparaffinized in ethanol-xylene series, followed by antigen retrieval for 15 min in Tris-EGTA buffer (10mM, pH 9.0, 90°C). Endogenous peroxidase activity was blocked for 10 min in 1% H_2_O_2_ + potassium phosphate buffered saline (KPBS) + 0.3% Triton-X (TX). Blocking of unspecific binding was obtained with 5% swine serum in KPBS + 0.25% TX + 1% BSA for 20 min followed by addition of mouse anti-β-amyloid primary antibody (Amyloid 82E1, IBL, Minneapolis, MN) diluted 1:500 in KPBS + 0.3% TX + 1% BSA for 45 min. After a rinse in buffer, sections were incubated for 30 min in Envision Polymer anti-mouse secondary antibody (Dako, Glostrup, Denmark). Sections were rinsed in buffer before subjected to 3,3′-diaminobenzidine as chromagen (Sigma-Aldrich, Broendby, Denmark). Development was stopped after 10 min with water before counterstaining for 30 seconds with Mayer’s haematoxylin (Dako, Glostrup, Denmark) diluted 1:3. Slides were mounted with Pertex and allowed to dry overnight before being scanned on a digital slide scanner (Aperio ScanScope AT, Leica Biosystems, Ballerup, Denmark). Brain atrophy and stereological assessment of volume fractions was performed in all animals in relevant brain areas of hAPP_Lon_/hPS1_A246E_, hAPP_Swe_/hPS1_ΔE9_ mice as well as in the corresponding wild-type littermate controls. These areas included frontal and caudal cortex (anterior and posterior to corpus callosum), hippocampus, striatum (caudate putamen, anterior commisure, nucleus accumbens), cerebellum and other gray matter (thalamus, ventral pallidum, medial forebrain bundle, septal nucleus, hypothalamus, substantia nigra and ventral tegmental area). The volume fractions of amyloid plaque formations were assessed in the same areas. The brain regions of interest were selected based on an introductory screening of brain regions in transgenic mice significantly affected by β-amyloid plaque load. The brain regional β-amyloid plaque volume and corresponding total regional volume was estimated by point counting (Cavalieri principle) using the following equation: Vol = ∑ P x a(P) x (1/ssf) x T, according to Gundersen *et al*. (1988) [49], with P referring to the number of points hitting the structure, a(P) is the area per point, ssf is the section sampling fraction and T is the thickness of the sections. As only one hemisphere was used for stereological estimates, the data were multiplied by two to obtain a total estimate for the whole brain. The regional brain areas were estimated using a 4-point grid whereas the regional amyloid plaque volumes were estimated using a 25-point grid at approximately 350x magnification providing a coefficient of error of 0.10, an ample precision compared to the observed biological variance [50].

### Data analysis

All data were fed into Excel spread sheets and subsequently subjected to relevant statistical analyses using GraphPad Prism 5.0. Results are presented as mean ± standard error of the mean (S.E.M.). Statistical evaluation of the data was carried out using a one-way analysis of variance (ANOVA; drug exposure, probe trial in the MWM, brain volume, plaque volume), or a repeated-measure two-way ANOVA (time x treatment; body weight, food intake, task acquisition in MWM, novel object recognition, T-maze task) with appropriate post-hoc analyses (one-way ANOVA, Dunnet’s test; two-way ANOVA, Bonferroni’s test) between control and treatment groups in cases where overall statistical significance was established. A p-value less than 0.05 was considered statistically significant.

## Results

### Body weight gain and survival rate

Liraglutide treatment was well-tolerated in transgenic hAPP_Lon_/PS1_A246E_ and hAPP_Swe_/PS1_ΔE9_ female mice. In general, both vehicle-dosed and liraglutide-treated transgenic mice showed normal body weight gain, as compared to corresponding age-matched vehicle-dosed wild-type mice.

#### Transgenic hAPP_Lon_/PS1_A246E_ mice

**Fig 1:**
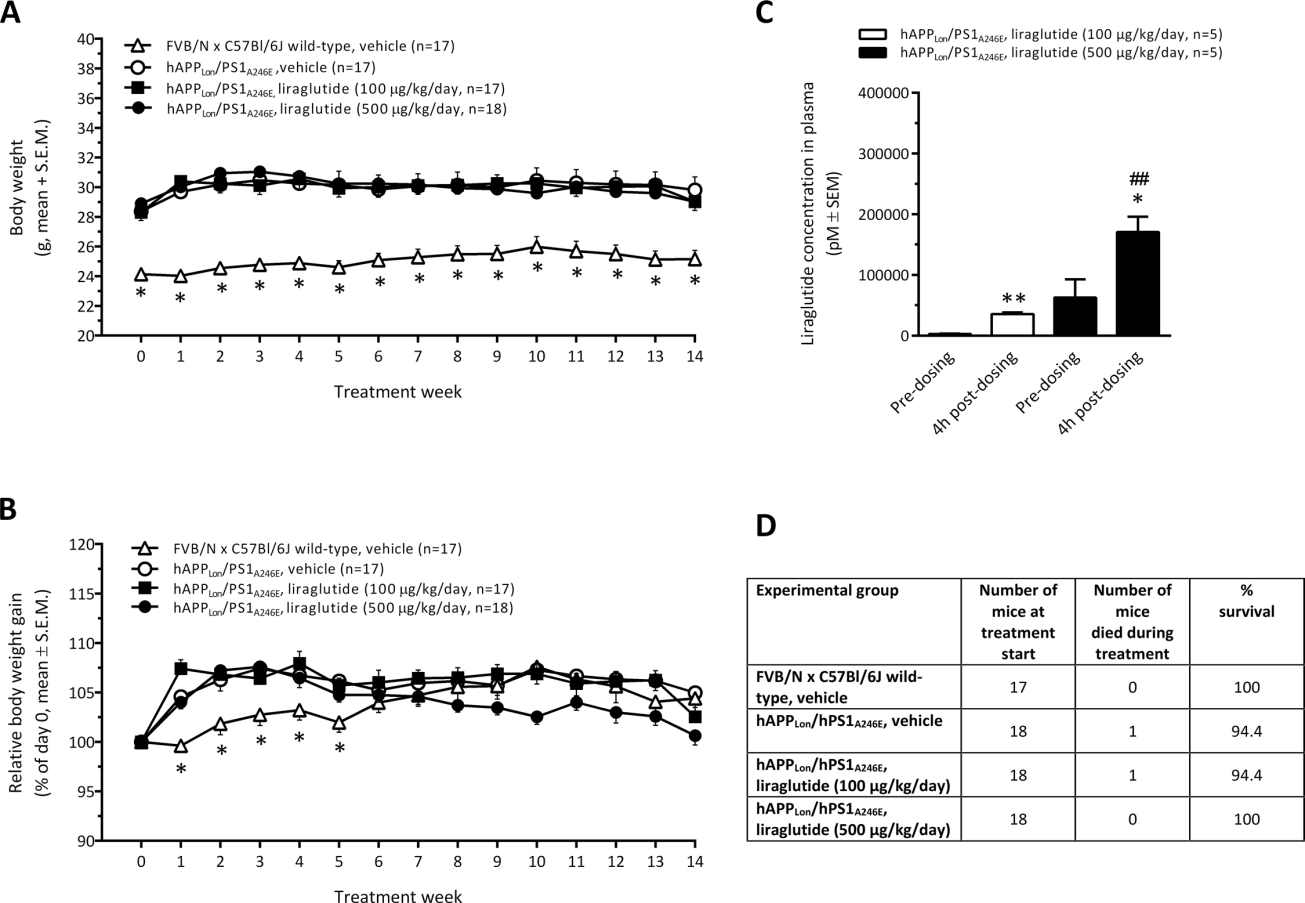
Transgenic hAPP_Lon_/PS1_A246E_ mice. (A) Absolute body weight development; (B) Relative body weight development (% of day 0). *p<0.05 vs. individual experimental groups of hAPP_Lon_/PS1_A246E_ mice; (C) Plasma liraglutide concentrations determined on the same day after 1½ month of dosing (pre-dosing vs. 4h post-dosing). *p<0.05, **p<0.01 vs. pre-dosing, ^##^p<0.01 vs. liraglutide (100 ng/kg/day); (D) Survival rate of wild-type FVB/N x C57Bl/6J controls and hAPP_Lon_/PS1_A246E_ mice.

Vehicle-dosed FVB/N wild-type control mice weighed significantly less than their transgenic hAPP_Lon_/PS1_A246E_ counterparts (p<0.001), initially amounting to a mean body weight difference of approximately 4 grams, with the absolute body weight difference kept stable throughout the 3-month dosing period (Fig 1A). Irrespective of treatment, the initial body weight gain of transgenic hAPP_Lon_/PS1_A246E_ mice was significantly higher within the first five weeks of treatment (p<0.05), as compared to female wild-type controls, whereupon the body weight remained stable in all experimental groups (Fig 1B). As compared to vehicle-dosed transgenic hAPP_Lon_/PS1_A246E_ mice, the highest dose of liraglutide (500 μg/kg/day) tended to reduce relative body weight gain in transgenic hAPP_Lon_/PS1_A246E_ mice, however, without attaining statistical significance (p = 0.119). Analysis of plasma liraglutide concentrations sampled 1½ month after dosing start confirmed drug exposure in all liraglutide-treated transgenic hAPP_Lon_/PS1_A246E_ mice. Liraglutide levels were significantly and dose-dependently elevated at 4 hours post-dosing vs. pre-dosing levels determined on the same sampling day (Fig 1C). As compared to vehicle-dosed wild-type control mice, both vehicle-dosed and liraglutide-treated transgenic hAPP_Lon_/PS1_A246E_ mice showed normal endpoint survival rate (Fig 1D).

#### Transgenic hAPP_Swe_/PS1_ΔE9_ mice

**Fig 2:**
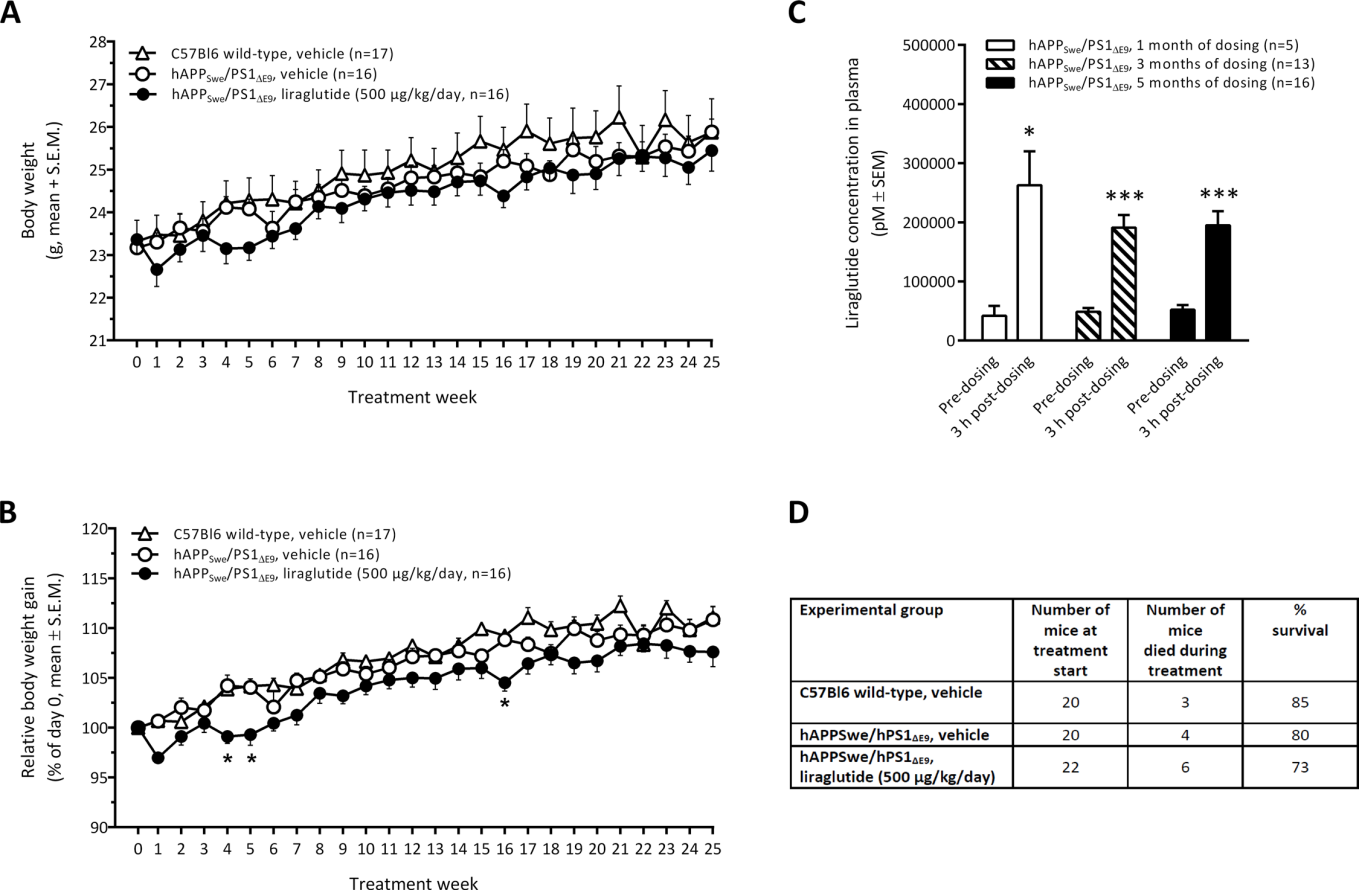
Transgenic hAPP_Swe_/PS1_ΔE9_ mice. (A) Absolute body weight development; (B) Relative body weight development (% of day 0). *p<0.05 vs. individual experimental groups of hAPP_Lon_/PS1_A246E_ mice; (C) Plasma liraglutide concentrations determined after 1, 3 and 5 months of daily dosing (pre-dosing vs. 3h post-dosing). *p<0.05, ***p<0.001 vs. pre-dosing; (D) Survival rate of C57Bl6 wild-type controls and hAPP_Swe_/PS1_ΔE9_ mice.

Seven-month old transgenic hAPP_Swe_/PS1_ΔE9_ and age-matched C57Bl6 wild-type mice had equal mean body weight at treatment start. Overall, all experimental groups showed similar body weight development throughout the 5 month dosing period (Fig 2A). While both genotypes dosed with vehicle had almost overlapping relative body weight gain curves, liraglutide-treated (500 μg/kg/day) transgenic hAPP_Swe_/PS1_ΔE9_ mice showed a slight, however consistent, reduction in body weight gain amounting to about 2% as compared to vehicle-dosed controls (overall p = 0.004, Fig 2B). Sustained systemic exposure of liraglutide was confirmed in plasma samples obtained after 1, 3 and 5 months of dosing with significant elevations of plasma liraglutide concentrations 4 hours after dosing, as compared to the corresponding pre-dosing level (Fig 2C). Upon completion of the treatment period (mice being 12 months of age), the endpoint survival rate of vehicle-dosed C57Bl6 wild-type controls was 85% (Fig 2D). Vehicle-dosed and liraglutide-treated APP_Swe_/PS1_ΔE9_ mice had an endpoint survival rate of 80% and 73%, respectively. Endpoint survival rate of wild-type and APP_Swe_/PS1_ΔE9_ mice, irrespectively of treatment, was not statistically significant (chi square p = 0.441, Mantel-Cox test).

### Memory performance

#### Transgenic hAPP_Lon_/PS1_A246E_ mice

**Fig 3:**
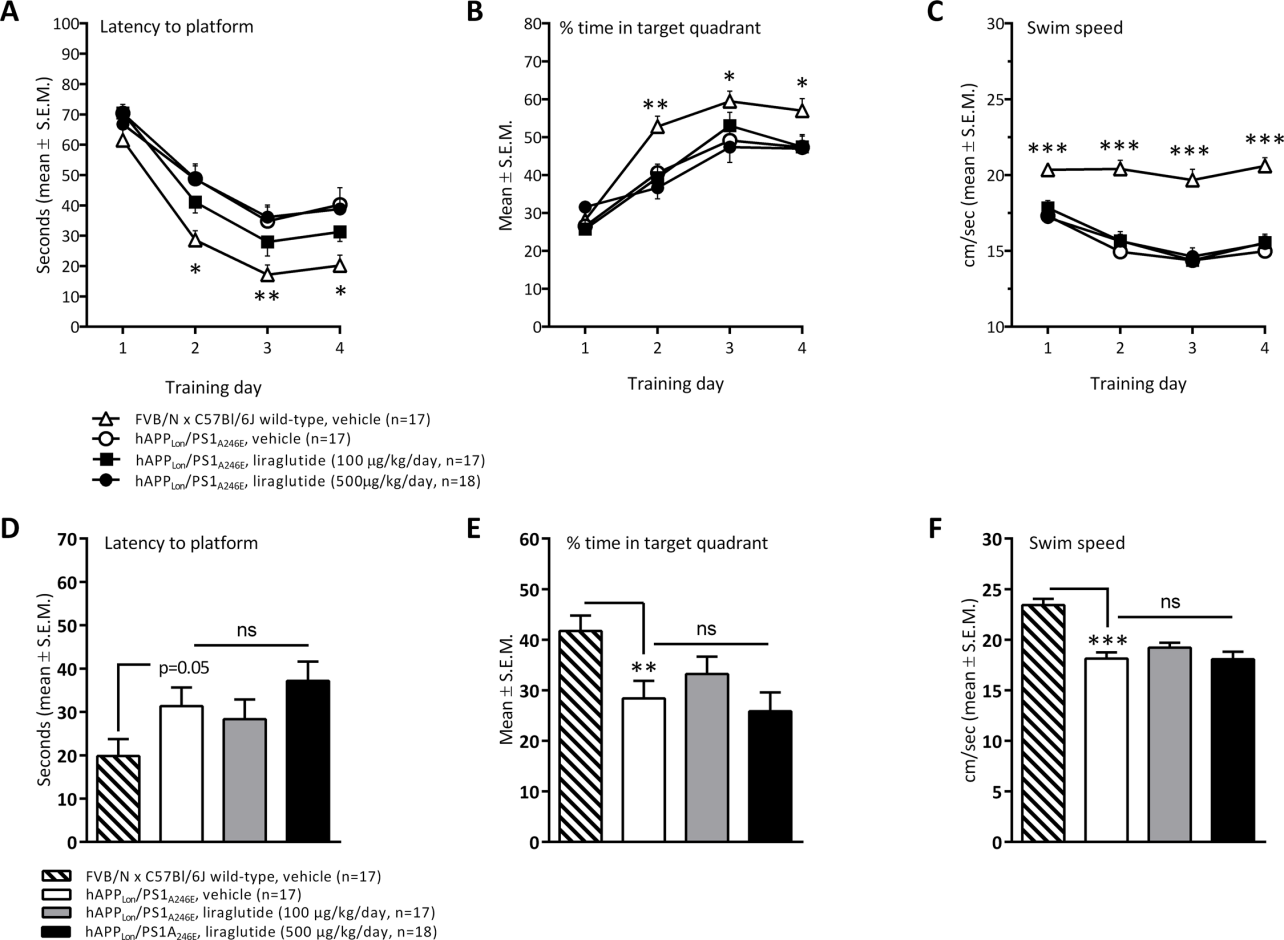
Transgenic hAPP_Lon_/PS1_A246E_ mice. Memory performance assessed in a Morris water maze task. Trial acquisition (training): (A) latency to platform, (B) % time spent in target quadrant, (C) swim speed. Spatial reference memory (probe trial): (D) latency to platform, (E) % time spent in target quadrant, (F) swim speed. **p<0.01, ***p<0.001 (wild-type FVB/N x C57Bl/6J controls vs. vehicle-dosed hAPP_Lon_/PS1_A246E_ mice).

As compared to age-matched FVB/N wild-type controls, vehicle-dosed 8 month-old transgenic hAPP_Lon_/PS1_A246E_ mice spent significant more time to reach the platform during MWM trial acquisition, as indicated by a significantly increased latency time (overall p<0.001, repeated-measure two-way ANOVA) during training day 2 (p<0.001), day 3 (p<0.01), and day 4 (p<0.001), see Fig 3A. hAPP_Lon_/PS1_A246E_ mice also exhibited significantly less time spent in target quadrant (overall p<0.001, repeated-measure two-way ANOVA) on the same training days (training day 2, p<0.01; day 3, p<0.05); day 4,p<0.05), see Fig 3B. As compared to wild-type controls, APP_Lon_/PS1_A246E_ mice showed slightly lower swim speed in both the task acquisition and probe trial (Fig 3C and 3F). Compared to wild-type control mice, transgenic mice receiving a daily dose of 100 ng liraglutide/kg/day, but not 500 ng/kg/day, exhibited no significant differences (p<0.05) in time to reach the platform (all training days) and percent time spend in the target quadrant (training days 3 and 4). A more stringent statistical analysis (repeated-measure two-way ANOVA, only including hAPP_Lon_/PS1_A246E_ experimental groups), indicated that vehicle-dosed and liraglutide-treated hAPP_Lon_/PS1_A246E_ mice, irrespectively of dose, showed no significant difference on task acquisition parameters in the MWM (latency to platform, overall p = 0.2470; % time in target quadrant, overall p = 0.9575), see Fig 3A and 3B.

When subjected to a probe trial on day 5, APP_Lon_/PS1_A246E_ mice had a significantly lower performance, compared to age-matched wild-type controls, as indicated by significantly less time spent in the target quadrant (t-test, p = 0.006, Fig 3E). APP_Lon_/PS1_A246E_ mice showed a tendency to increase latency time to reach the platform area (t-test, p = 0.056, Fig 3D). Probe trial performance was equal in vehicle-dosed and liraglutide-treated hAPP_Lon_/PS1_A246E_ mice, as indicated by similar latency time to reach the platform area (overall p = 0.353, one-way ANOVA, Fig 3D) and percent time spent in target quadrant (overall p = 0.336, one-way ANOVA, Fig 3E).

#### Transgenic hAPP_Swe_/PS1_ΔE9_ mice

**Fig 4:**
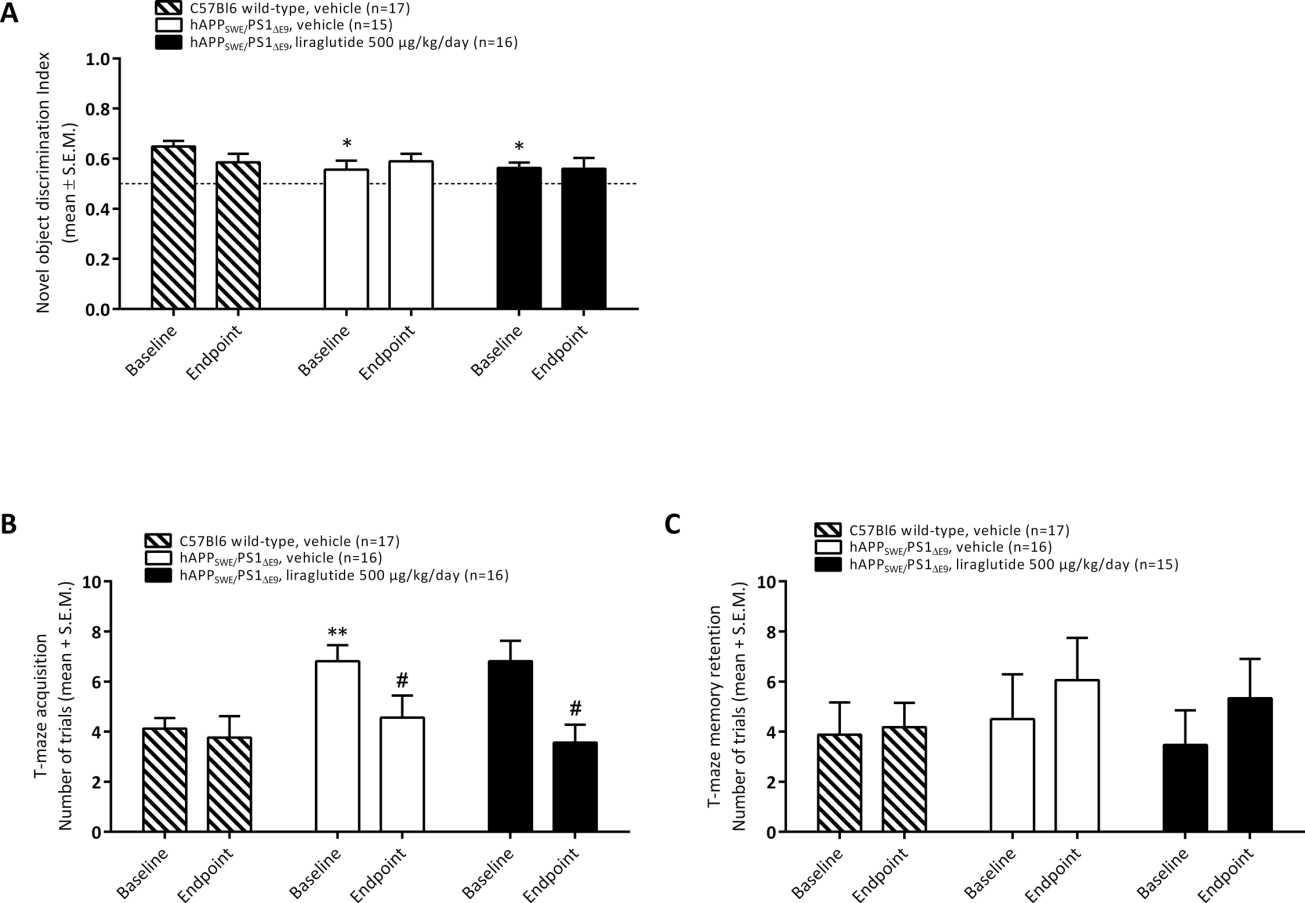
Transgenic hAPP_Swe_/PS1_ΔE9_ mice. Memory performance assessed in a novel object recognition (A) and active-avoidance T-maze (B, C) task, respectively. (A) Novel object recognition index, (B) Number of trials in T-maze acquisition trial; (C) Number of trials in T-maze memory retention trial. *p<0.05 (vs. wild-type control), ^#^p<0.05 (vs. baseline performance).

In general, data obtained in the NOR test showed less variability, as compared to performance in the active avoidance T-maze task (Fig 4). Baseline NOR and T-maze memory performance was assessed in 6½ month-old transgenic hAPP_Swe_/PS1_ΔE9_ mice prior to initiation of 5 month of treatment with liraglutide. As compared to age-matched wild-type C57Bl6 control mice (n = 17), hAPP_Swe_/PS1_ΔE9_ mice (n = 31) showed significantly reduced object discrimination index in the NOR test (0.65 ± 0.02 vs. 0.56 ± 0.02, p = 0.008, unpaired t-test). 7 month-old hAPP_Swe_/PS1_ΔE9_ mice also showed impaired baseline performance in the active avoidance T-maze task, as indicated by a significantly (p = 0.001, unpaired t-test) increased number of trials to complete the acquisition trial in hAPP_Swe_/PS1_ΔE9_ mice (6.8 ± 0.5 trials, n = 32) as compared to wild-type controls (4.1 ± 0.4 trials, n = 20). The number of trials to reach criterion in the T-maze memory retention trial were similar (p = 0.948, unpaired t-test) in 7 month-old wild-type mice (3.8 ± 1.3 trials, n = 20) and hAPP_Swe_/PS1_ΔE9_ mice (4.0 ± 1.1 trials, n = 42). No gross motor deficits were observed in hAPP_Swe_/PS1_ΔE9_ mice at baseline memory assessment. Upon completion of the T-maze task, hAPP_Swe_/PS1_ΔE9_ mice were subsequently randomized to receive either vehicle-dosing (n = 20) or liraglutide treatment (n = 22) once daily over 5 months, by balancing mean baseline object discrimination index (0.56 ± 0.04 vs. 0.56 ± 0.02, p = 0.876), T-maze acquisition (6.8 ± 0.6 vs. 6.8 ± 0.8 trials, p>0.999) and T-maze retention (4.5 ± 1.8 vs. 3.8 ± 1.3 trials, p = 0.655) in the two groups. Upon randomization, both hAPP_Swe_/PS1_ΔE9_ groups exhibited significant impairments in object recognition index (overall p = 0.030, one-way ANOVA; p<0.05 for both groups vs. wild-type control, Dunnett’s post-hoc test) and T-maze acquisition (overall p = 0.004, one-way ANOVA; p<0.01 for both groups vs. wild-type control, Dunnett’s post-hoc test), see Fig 4A and 4B.

At completion of 5 months of treatment, surviving wild-type and transgenic mice (vehicle-dosed wild-type mice, n = 17; vehicle-dosed hAPP_Swe_/PS1_ΔE9_ mice, n = 16; liraglutide-treated hAPP_Swe_/PS1_ΔE9_ mice, n = 16; see Fig 2D) were re-evaluated for memory performance in both the NOR and T-maze tasks, respectively (Fig 3). For comparison to baseline memory performance, only mice surviving at study termination were included in the data analyses. As compared to baseline, endpoint object discrimination index was unaltered (overall p = 0.359, repeated-measure two-way ANOVA, time x treatment) in the experimental groups. Vehicle-dosed wild-type control mice tended to exhibit reduced object endpoint discrimination index, although this did not attain statistical significance (0.65 ± 0.02 vs. 0.59 ± 0.03, p = 0.164). In contrast, NOR performance remained very stable in vehicle-dosed hAPP_Swe_/PS1_ΔE9_ mice (0.56 ± 0.03 vs. 0.59 ± 0.03, p = 0.502), and liraglutide-treated hAPP_Swe_/PS1_ΔE9_ mice (0.56 ± 0.02 vs. 0.56 ± 0.04, p = 0.952) see Fig 4A. Endpoint recognition index (time spent at novel minus total exploration time of both objects) hAPP_Swe_/PS1_ΔE9_ mice was also unchanged by liraglutide treatment, as compared to vehicle-dosed control and hAPP_Swe_/PS1_ΔE9_ mice, respectively (data not shown).

Evaluation of baseline vs. endpoint of T-maze acquisition performance indicated no change in the number of trials to the complete task in wild-type control mice (4.1 ± 0.4 vs 3.8 ± 0.9 trials, p>0.05, repeated-measure two-way ANOVA). In contrast, when comparing to baseline performance the number of endpoint acquisition trials in hAPP_Swe_/PS1_ΔE9_ mice was significantly lower after 5 months of dosing of vehicle (6.8 ± 0.6 vs 4.6 ± 0.9 trials, p<0.05) and liraglutide (6.8 ± 0.8 vs 3.6 ± 0.7 trials, p<0.05), respectively. Hence, all experimental groups showed similar endpoint T-maze acquisition (p = 0.137, repeated-measure two-way ANOVA, time x treatment) at study termination, see Fig 4B. T-maze memory retention was unaltered (p = 0.3590, repeated-measure two-way ANOVA, time x treatment) in wild-type controls (3.8 ± 1.3 vs 4.2 ± 1.0 trials), as well as vehicle-dosed (4.5 ± 1.3 vs 6.1 ± 1.7 trials) and liraglutide-treated hAPP_Swe_/PS1_ΔE9_ mice (3.8 ± 1.3 vs 6.8 ± 2.1 trials).

### β-amyloid plaque load

**Fig 5:**
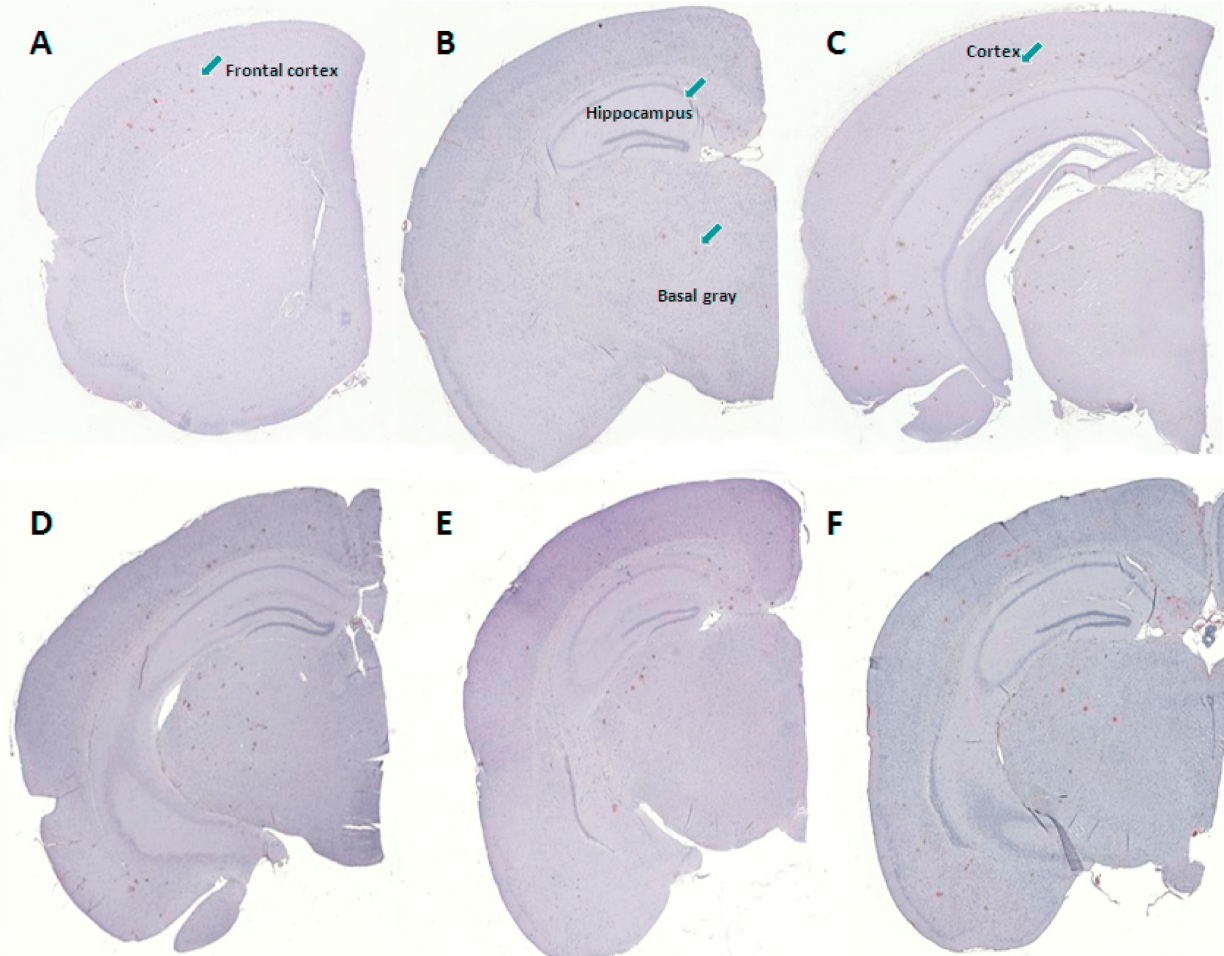
Representative photomicrographs depicting β-amyloid plaque levels in eight month-old transgenic hAPP_Lon_/PS1_A246E_ mice. (A-C) Plaque depositions in vehicle-dosed hAPP_Lon_/PS1_A246E_ mice, indicated at three individual coronal levels. (D) vehicle-dosed hAPP_Lon_/PS1_A246E_ mouse (E) liraglutide-treated hAPP_Lon_/PS1_A246E_ mouse (100 μg/kg/day); (F) liraglutide-treated hAPP_Lon_/PS1_A246E_ mouse (500 μg/kg/day).

**Fig 6:**
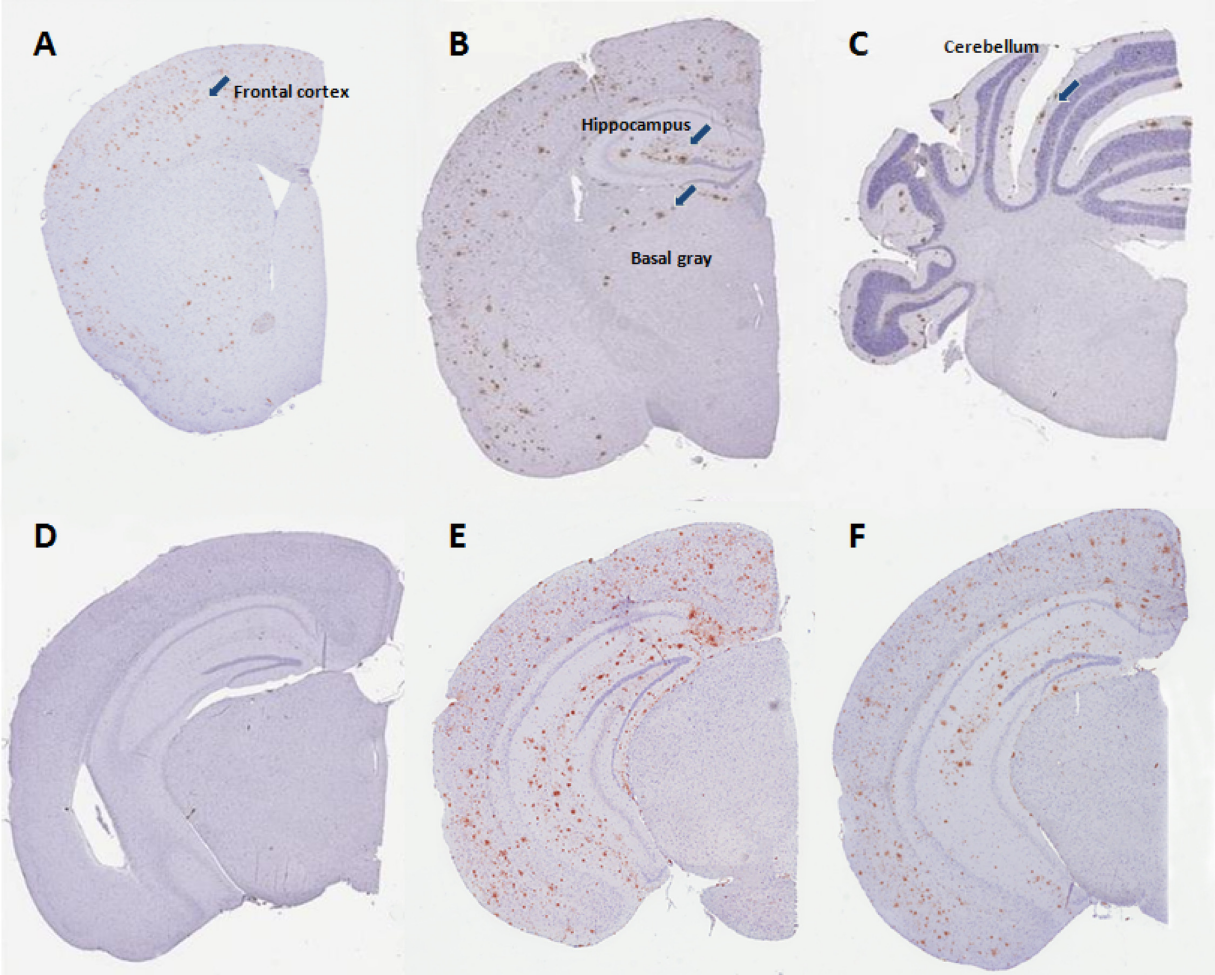
Representative photomicrographs depicting β-amyloid plaque levels in 12 month-old transgenic hAPP_Swe_/PS1_ΔE9_ mice. (A-C) Plaque depositions in vehicle-dosed hAPP_Swe_/PS1_ΔE9_ mice, indicated at three individual coronal levels. (D) vehicle-dosed wild-type mouse, (E) vehicle-dosed hAPP_Swe_/PS1_ΔE9_ mouse, (F) liraglutide-treated hAPP_Swe_/PS1_ΔE9_ mouse (500 μg/kg/day).

A qualitative screening of β-amyloid immunoreactivity was performed throughout the entire brain of vehicle-dosed hAPP_Lon_/PS1_A246E_ mice and hAPP_Swe_/PS1_ΔE9_ mice, respectively, to identify brain regions showing presence of β-amyloid plaques. Representative photomicrographs of relevant immunostained brain regions are shown in Fig 5 (hAPP_Lon_/PS1_A246E_ mice) and Fig 6 (hAPP_Swe_/PS1_ΔE9_ mice).

#### Transgenic hAPP_Lon_/PS1_A246E_ mice

**Fig 7:**
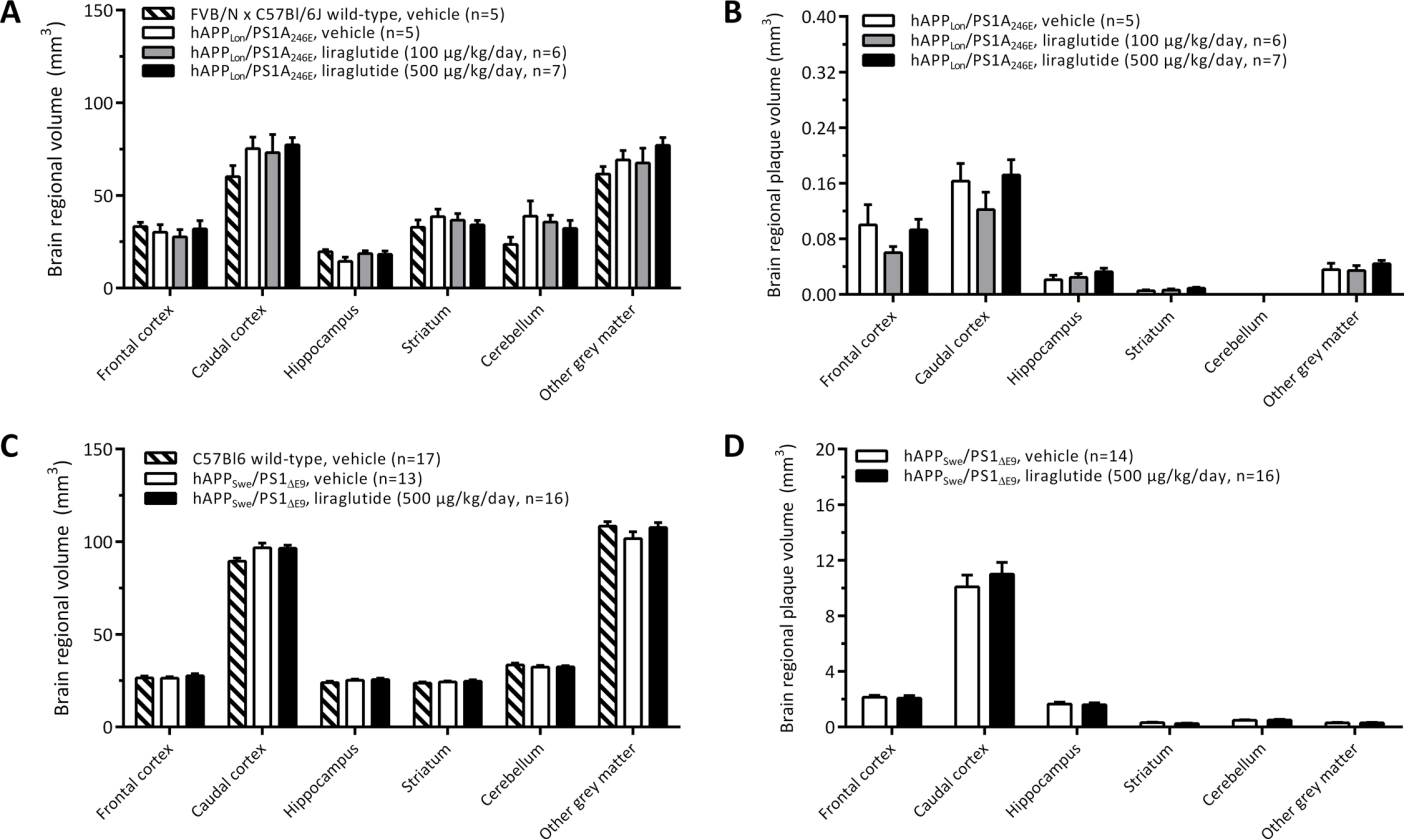
Stereological analysis of brain regional volume and plaque volume in transgenic hAPP_Lon_/PS1_A246E_ and hAPP_Swe_/PS1_ΔE9_ mice. Stereological assessments were only performed in brain structures showing presence of β-amyloid plaques. Liraglutide treatment had no effect on brain regional volume and plaque load in hAPP_Lon_/PS1_A246E_ (A, B) and hAPP_Swe_/PS1_ΔE9_ (C, D) mice.

Total brain and regional volumes were similar in 8 month-old transgenic hAPP_Lon_/PS1_A246E_ and corresponding age-matched wild-type controls, indicating no brain atrophy in hAPP_Lon_/PS1_A246E_ mice (Fig 7A). The stereological quantitative analysis of β-amyloid immunoreactive volumes indicated low abundance of β-amyloid plaques in the brains of vehicle-dosed hAPP_Lon_/PS1 _A246E_ mice, with the total plaque volume amounting to approximately 0.1% of total brain volume (Table 1). In hAPP_Lon_/PS1_A246E_ mice, β-amyloid plaques were present in the frontal cortex, caudal cortex, hippocampus, striatum and other gray matter, but not in the cerebellum (Figs 5 and 7B). Age-matched vehicle-dosed wild-type control mice did not show evidence of β-amyloid immunoreactivity in any brain region examined. As compared to vehicle dosing, liraglutide treatment did not influence plaque volumes in any brain region affected in hAPP_Lon_/PS1 _A246E_ mice (p>0.05 for all brain regions examined, Fig 7B). This also applied when summating total plaque volumes as well as calculating the plaque load relative to total brain volume (Table 1).

**Table 1:** Total brain volume and plaque levels in two transgenic hAPP/PS1 mouse models of Alzheimer’s disease, as determined by stereological means.

Experimental group	Total brain volume (mm^3^)	Total plaque volume (mm^3^)	Total relative plaque load (% of total brain volume)
FVB/N wild-type, vehicle	233.8 ± 15.5	n/a	n/a
hAPP_LON_/PS1_A246E_, vehicle	268.8 ± 13.2	0.324 ± 0.058	0.118 ± 0.014
hAPP_LON_/PS1_A246E_, liraglutide (100 μg/kg/day)	272.9 ± 17.0	0.246 ± 0.040	0.095 ± 0.010
hAPP_LON_/PS1_A246E_, liraglutide (500 μg/kg/day)	261.3 ± 24.0	0.352 ± 0.044	0.127 ± 0.012
C57Bl6 wild-type, vehicle	305.6 ± 4.3	n/a	n/a
hAPP_SWE_/PS1_ΔE9_, vehicle	306.4 ± 6.5	14.98 ± 1.08	4.98 ± 0.40
hAPP_SWE_/PS1_ΔE9_, liraglutide (500 μg/kg/day)	314.5 ± 4.7	15.72 ± 1.14	5.01 ± 0.36

Eight-month old transgenic hAPP_LON_/PS1_A246E_ control mice displayed very mild amyloidosis. In contrast, 12-month old transgenic hAPP_SWE_/PS1_ΔE9_ control mice exhibited a severe beta-amyloid plaque burden (approximately 40-fold greater than hAPP_LON_/PS1_A246E_ mice). Long-term liraglutide treatment did not influence total brain volume, β-amyloid plaque volume and relative beta-amyloid plaque load in transgenic hAPP_LON_/PS1_A246E_ and hAPP_SWE_/PS1_ΔE9_ mice, as compared to vehicle treatment (p>0.05 for all parameters compared). n/a, not applicable.

#### Transgenic hAPP_Swe_/PS1_ΔE9_ mice

Total brain and regional volumes were similar in 12 month-old transgenic hAPP_Swe_/PS1_ΔE9_ mice and corresponding C57Bl6 wild-type controls, *i*.*e*. no gross brain atrophy was observed in hAPP_Swe_/PS1_ΔE9_ mice (Fig 7C, Table 1). The total brain volume was significantly greater (approximately 14%) in vehicle-dosed hAPP_Swe_/PS1_ΔE9_ mice, as compared to vehicle-dosed hAPP_Lon_/PS1 _A246E_ mice (p = 0.012, unpaired t-test). Age-matched C57Bl6 wild-type control mice did not show evidence of β-amyloid immunoreactivity in any brain region examined. In contrast to hAPP_Lon_/PS1_A246E_ mice, hAPP_Swe_/PS1_ΔE9_ mice exhibited a rather similar brain regional distribution of plaques (caudal cortex > frontal cortex > hippocampus >> striatum), however the plaque load was considerably higher in hAPP_Swe_/PS1_ΔE9_ mice (compare Figs 5 and 6). Accordingly, hAPP_Swe_/PS1_ΔE9_ mice showed a total plaque volume load being approximately 40 times higher than hAPP_Lon_/PS1_A246E_ mice, in particular with the cortical regions and hippocampus being markedly more affected in hAPP_Swe_/PS1_ΔE9_ (Fig 7D, Table 1). Vehicle-dosed and liraglutide-treated hAPP_Swe_/PS1_ΔE9_ mice exhibited very similar brain regional and total β-amyloid plaque volumes (p>0.05 for all brain regions examined, Fig 7D, Table 1).

## Discussion

The present study compared effects of long-term liraglutide administration in two transgenic mouse models of AD, representing a phenotype of low-grade (hAPP_Lon_/PS1_A246E_ mice) and high-grade (hAPP_Swe_/PS1_ΔE9_ mice) amyloidosis, respectively.

Immunohistochemical determination of β-amyloid expression indicated that 8 month-old hAPP_Lon_/PS1_A246E_ and 12 month-old hAPP_Swe_/PS1_ΔE9_ mice displayed similar brain regional distribution of β-amyloid plaque deposits with highest plaque levels found in cortical regions and the hippocampus, being consistent with previous findings [30,36–38,51]. In line with previous reports demonstrating no gross atrophy or neuronal loss in severely plaque-affected brain regions of double transgenic APP/PS1 mouse models [27,44], brain regional volumes were similar in hAPP_Lon_/PS1_A246E_ and hAPP_Swe_/PS1_ΔE9_ mice, as compared to their corresponding wild-type controls. The comprehensive stereological analysis also indicated that hAPP_Swe_/PS1_ΔE9_, as compared to hAPP_Lon_/PS1_A246E_ mice, had markedly higher plaque volumes (>40-fold) in all relevant brain regions examined. Although it should be taken into account that the comparative stereological analyses were not applied to age-matched hAPP_Lon_/PS1_A246E_ and hAPP_Swe_/PS1_ΔE9_ mice, the age-difference between the two transgenic APP/PS1 mouse models does not explain the substantial differences in plaque load. hAPP_Lon_/PS1_A246E_ mice present with cortical and hippocampal Aβ42 overexpression, increased ratios of soluble Aβ42:Aβ40 and corresponding plaque formation at 6 months of age with a relatively slow plaque growth rate, as pronounced plaque burden is observed only at the age of 20 months [30,37,38]. In contrast, hAPP_Swe_/PS1_ΔE9_ mice display earlier onset and accelerated cerebral amyloidosis with disseminating and severe plaque burden being evident at the age of 12–14 months [36,44–46,51–53]. Consequently, it is more likely that the marked difference in plaque load is associated with the different hAPP/PS1 transgenic mouse model genotypes where particularly the PS1_ΔE9_ mutation, as compared to the PS1_A246E_ variant, promotes much higher steady-state levels of Aβ42 with corresponding higher degree of plaque aggregation [36].

To account for differential progression rates of amyloidosis in the two AD models, liraglutide treatment was initiated in both models at individual ages when only very low-grade amyloidosis would be expected. Hence, liraglutide administration was started when hAPP_Lon_/PS1_A246E_ (3 months of treatment) and hAPP_Swe_/PS1_ΔE9_ mice (5 months of treatment) were 5 and 7 month-old, respectively. Application of a low (100 μg/kg/day, equivalent to 26 nmol/kg/day) or moderately high (500 μg/kg/day, equivalent to 133 nmol/kg/day) dose of liraglutide resulted in dose-dependent systemic drug exposure in hAPP_Lon_/PS1_A246E_ mice, as determined 1½ month after treatment start. The moderately high dose of liraglutide resulted in similar plasma levels in both hAPP_Lon_/PS1_A246E_ and hAPP_Swe_/PS1_ΔE9_ mice, and further intermittent blood sampling in hAPP_Swe_/PS1_ΔE9_ mice indicated sustained systemic liraglutide exposure throughout the whole treatment period. Similar to other inbred or transgenic mouse models of AD [34,35,54], long-term GLP-1 receptor agonist treatment evoked only very modest or no weight-lowering effects in hAPP_Swe_/PS1_ΔE9_ mice. As GLP-1 receptor agonists, including exendin-4 and liraglutide, exhibit much greater body weight lowering and anorectic effects in obese mice [55–57], this likely explains the very mild and short-lasting metabolic effects of long-term liraglutide treatment in the present study. Remarkably, the hAPPLon/PS1A246E mice weighed less than the control mice on an identical genetic background, despite the fact that several APP and APP/PS1 lines exist that have a lower weight than wild-type controls. Although this could be the result of separate breeding of the controls, this is unlikely. The APP parental line is bred heterozygously and the PS1 line although bred homozygously is occasionally crossed back with wild-type mice to maintain a stable genetic background. The reduced weight of the double transgenic mice has been found consistently in different studies. These APP_Lon_/PS1_A246E_ mice have been shown amenable to cognitive improvement by Aβ targeting compounds [41,58]

Because quantification of β-amyloid plaque levels is one of the primary endpoints of AD model characterization, it is therefore also critical for assessment of neuroprotective effects of putative therapeutic drugs. In this regard, stereological analysis is considered the optimal tool for quantitative assessment of morphological changes and treatment interventions in neurodegeneration models, as this method is based on random sampling techniques and generation of unbiased estimates of three-dimensional characteristics [49,59,60]. We have recently reported comprehensive stereological studies on liraglutide’s neuroprotective effects in a non-transgenic mouse model of age-related AD as well as in a transgenic mouse model of tauopathy [34,35]. In contrast, similar comparable stereological analyses have not been applied for in-depth histological evaluation of GLP-1 receptor agonist treatment in transgenic hAPP/PS1 mouse models of AD. In conjunction with assessment of memory function in hAPP_Lon/_PS1_A246E_ and hAPP_Swe_/PS1_ΔE9_ mice, stereological methods were therefore employed for comprehensive mapping of β-amyloid plaques levels throughout the brain after long-term liraglutide treatment in the two transgenic models.

The stereological analyses demonstrated that long-term liraglutide treatment had no effect on β-amyloid plaque load and corresponding tissue volumes across all brain regions examined in 8 month-old hAPP_Lon_/PS1_A246E_ and 12 month-old hAPP_Swe_/PS1_ΔE9_ mice, respectively. GLP-1 receptor agonists have previously been evaluated for potential plaque-lowering properties in transgenic mouse models of AD, as summarized in Table 2. In contrast to our findings, GLP-1 receptor agonists are reported to reduce markers of β-amyloid toxicity in a comparable transgenic hAPP_Swe_/PS1_ΔE9_ mouse model. Accordingly, exendin-4 administration reduced cortical plaque load and soluble β-amyloid levels in hAPP_Swe_/PS1_ΔE9_ male mice [21]. Longer-acting GLP-1 receptor agonists have also been reported to promote plaque-lowering effects in a similar hAPP_Swe_/PS1_ΔE9_ transgenic mouse strain. Accordingly, administration of liraglutide (25 nmol/kg/day, equivalent to 94 μg/kg/day) reduces cortical and hippocampal plaque accumulation at different ages in both female and male hAPP_Swe_/PS1_ΔE9_ mice [23,25,61,62]. Also a lower dose of liraglutide (2.5 nmol/kg/day), as well as lixisenatide administration (1–10 nmol/kg/day), is reported being efficacious in reducing cortical plaque burden in this transgenic mouse strain [22].

**Table 2:** Studies characterizing the effect of GLP-1 receptor agonists and other related compounds in different transgenic mouse models on β-amyloid plaque pathology.

Transgenic mouse model	Plaque onset in model	Drug	Dosage	Gender (n)	Treatment start (age)	Treatment duration	Effect on plaque load	Ref.
APP_Swe_/PS1_ΔE9_	4–6 mo	Exendin-4	25 nmol/kg/day, i.p.	Male (n = 6)	13–14 months	3 weeks	Reduced plaque load (cortex)	[21]
APP_Swe_/PS1_ΔE9_	4–6 mo	Sitagliptin Exendin-4	20 mg/kg/day, p.o. 2 pM/kg/min, s.c. osmotic minipump	Male (n = 6)	7 months	12 weeks	Reduced plaque load (sitagliptin, hippocampus) No effect (exendin-4, hippocampus)	[63]
APP_Swe_/PS1_ΔE9_	4–6 mo	GLP-1(9–36)amide	151 nmol/kg/day, s.c. osmotic minipump	Male (n = 7)	10–12 months	2 weeks	No effect (hippocampus)	[65]
APP_Swe_/PS1_ΔE9_	4–6 mo	Liraglutide	25 nmol/kg/day, i.p.	Male (n = 6)	7 months	8 weeks	Reduced plaque load (hippocampus, cortex)	[23]
APP_Swe_/PS1_ΔE9_	4–6 mo	Liraglutide	25 nmol/kg/day, i.p.	Female (n = 5)	7 months	8 weeks	Reduced plaque load (cortex)	[61]
APP_Swe_/PS1_ΔE9_	4–6 mo	Liraglutide	25 nmol/kg/day, i.p.	Male (n = 12)	14 months	8 weeks	Reduced plaque load (cortex)	[25]
APP_Swe_/PS1_ΔE9_	4–6 mo	Liraglutide Lixisenatide	2.5–25 nmol/kg/day, i.p.1-10 nmol/kg/day, i.p.	Male (n = 11–12)	7 months	10 weeks	Reduced plaque load (cortex)	[22]
APP_Swe_/PS1_ΔE9_	4–6 mo	Liraglutide	25 nmol/kg/day, i.p.	Male (n = 6)	8 weeks	8 months	Reduced plaque load (cortex)	[62]
APP/PS1-21	2–3 mo	Val8(GLP-1)	25 nmol/kg/day, i.p.	Male (n = 11–15)	9 and 18 months	3 weeks	No effect (cortex)	[64]
APP_Swe_/PS1_M146V_/Tau_P301L_	6 mo	Exendin-4	3.5 pM/kg/min, s.c.osmotic minipump	Male (n = 3), Female (n = 4)	11–12.5 months	16 weeks	No effect (hippocampus, cortex)	[17]
APP_Swe_/PS1_M146V_/Tau_P301L_	6 mo	Exendin-4	119 nmol/kg/day, i.p. (5 days a week)	Male/female (n = 5)	3 months	9 months	No effect (hippocampus)	[54]

The transgenic mouse models of Alzheimer’s disease display age-dependent differences in the onset of plaque aggregation, i.e. APP_Swe_/PS1_ΔE9_, 4–6 month of age [36, 46]; APP/PS1-21, 2–3 month of age [66]; APP_Swe_/PS1_M146V_/Tau_P301L_, 6 months of age [67].

In contrast to these studies, others have not found plaque lowering effects of exendin-4 treatment in the hippocampus of hAPP_Swe_/PS1_ΔE9_ male mice [63]. Likewise, treatment with the GLP-1 analogue Val(8)GLP-1 did not reduce cortical plaque accumulation in a double APP/PS1-21 transgenic mouse model of AD [64]. Also, exendin-4 treatment showed no plaque-lowering effect in triple transgenic mice harbouring APP_Swe_/PS1_M146V_/Tau_P301L_ mutations [17,54].

In comparison, we have recently reported that long-term treatment regimens with liraglutide, using similar doses (100–500 μg/kg/day, s.c.) with equivalent dosing periods (4–5 months) and stereological measures, showed neuroprotective effects in two mouse models of AD without β-amyloid plaque pathology, i.e. non-transgenic SAMP8 mice [34] and hTauP301 transgenic mice [35].

The reason for the conflicting histological results in transgenic mouse models of β-amyloid overexpression is unclear, but the variable responsiveness to GLP-1 receptor agonist treatment may tentatively be explained by different experimental designs and methods employed. Hence, it is conceivable that the choice of mouse background strain, age and β-amyloid plaque level at treatment start, drug dose and duration of treatment as well as histological methods for quantitative assessment of plaque load are important determinants. It should be noted that liraglutide enters the murine brain upon systemic administration [4]. However, concentrations of liraglutide in mouse whole-brain extracts shows little dose-dependent differences when administered acutely in doses ranging from 2.5–250 nmol/kg, i.p. [68]. Similar observations have been reported for exendin-4 [69] and lixisenatide [68], indicating saturable central uptake of GLP-1 receptor agonists. Hence, although plaque levels were unaffected by administration of a low (hAPP_Lon_/PS1_A246E_ mice) or moderately high dose (hAPP_Lon_/PS1_A246E_ and hAPP_Swe_/PS1_ΔE9_ mice) of liraglutide, it cannot be ruled out that hAPP_Swe_/PS1_ΔE9_ mice could be responsive to lower doses of liraglutide.

In conjunction with stereological quantification of β-amyloid plaque levels, memory function was also evaluated in hAPP_Lon_/PS1_A246E_ and hAPP_Swe_/PS1_ΔE9_ mice. In this regard, it should be emphasized that different memory tests were applied to the two transgenic mouse lines, *i*.*e*. spatial reference memory was evaluated in hAPP_Lon_/PS1_A246E_ mice in a MWM task, whereas object and spatial reference memory was assessed in hAPP_Swe_/PS1_ΔE9_ mice in NOR and T-maze tests. Also, baseline performance was only evaluated in hAPP_Swe_/PS1_ΔE9_ mice, i.e. only hAPP_Swe_/PS1_ΔE9_ mice randomized to treatment based on pre-treatment memory performance. Hence, it is not possible to directly compare memory function in the two transgenic mouse models of AD.

While 8 month-old hAPP_Lon_/PS1_A246E_ controls showed increased latency to reach the platform and less time spent in target quadrant in both MWM training and probe trial, suggesting deficits in spatial learning and memory function, long-term liraglutide treatment in hAPP_Lon_/PS1_A246E_ mice did not affect these deficits in the MWM. hAPP_Lon_/PS1_A246E_ mice are previously reported to swim slower than wild-type controls [41]. The slightly lower swim speed may potentially have confounded latency data, but not probe trial data, and it should be emphasized that the probe trial represents the more stringent test for a spatial memory deficit. Moreover, an effect of donepezil on latency to platform has been shown in this AD model without affecting swim speed [41].

hAPP_Swe_/PS1_ΔE9_ mice exhibited impaired baseline memory performance in the NOR and T-maze task. The present T-maze task is considered an aversive learning-task based on hippocampus-dependent working and reference memory [70]. Novel object recognition also constitutes a declarative memory task that involves the hippocampus when, as performed here, the retention interval is 24 hours after initial exposure to the objects [71–73]. Hence, both tests indicate that 6½ month-old hAPP_Swe_/PS1_ΔE9_ mice exhibited impairments in hippocampus-associated memory function prior to initiation of long-term liraglutide treatment. Re-assessment of memory function in surviving 12-month old vehicle-dosed hAPP_Swe_/PS1_ΔE9_ mice indicated a very similar object discrimination index, as compared to baseline performance, in the transgenic controls. However, an apparent slight reduction in endpoint NOR performance of the wild-type control group, as well as a rather large within-group variability in the object discrimination index, may likely have precluded statistical evidence for sustained object memory deficit in vehicle-dosed hAPP_Swe_/PS1_ΔE9_ control mice at 12 months of age. Surprisingly, comparative analysis of baseline vs. endpoint T-maze performance indicated improved T-maze acquisition in the hAPP_Swe_/PS1_ΔE9_ controls, thus opposing rather stable T-maze acquisition in the corresponding wild-type control group. It should be emphasized that variations in pre-dosing memory task performance of hAPP_Swe_/PS1_ΔE9_ were accounted for by balancing both baseline NOR and T-maze performance in the treatment randomization procedure. Hence, the lack of detectable endpoint memory deficits in 12 month-old hAPP_Swe_/PS1_ΔE9_ mice cannot be ascribed to differences in mean baseline memory function. The age-dependent performance in the NOR and T-maze may potentially be related to different memory test designs used in other studies on GLP-1 receptor agonists. For example, a 24h inter-trial interval in the NOR test was applied on both test ages in the present study which must be considered a more demanding test paradigm, as compared to the much shorter (3h) inter-trial interval used in previous NOR studies reporting mnemonic effects of GLP-1 receptor agonists [22,23,25,62]. Using a 3h inter-trial interval, vehicle-dosed transgenic control mice showed reduced recognition index within this age interval, also when applying the test paradigm to the transgenic mice at both 7 and 9 months of age [23], indicating that repeated NOR tests might more consistently detect sustained deficits in short-term, rather than long-term, especially when testing memory function in relatively old hAPP_Swe_/PS1_ΔE9_ mice. This may also apply to assessment of GLP-1 receptor agonist effects in other mouse models of AD. In accordance, 10 month-old SAMP8 mice showed marked memory deficits in a similar active avoidance T-maze task design, whereas no memory deficits were apparent in a NOR test using a 24h inter-trial interval [34]. Whereas the aversive T-maze test paradigm has thus been applied to other mouse models of AD, the present study is to our knowledge the first to assess aversive T-maze based memory function in hAPP_Swe_/PS1_ΔE9_ mice. The lack of sustained learning deficits, as well as no change in both baseline and endpoint T-maze memory retention performance, suggests that the present T-maze paradigm is suboptimal in determining sustained memory deficits in this transgenic mouse model of AD.

Irrespective of the lack of impaired memory function in 12 month-old hAPP_Swe_/PS1_ΔE9_ mice, long-term liraglutide treatment had no effect on the marked cerebral plaque load in this transgenic model of AD. Similar lack of plaque-lowering efficacy of long-term liraglutide treatment was observed in eight month-old hAPP_Lon_/PS1_A246E_, consistent with the absence of mnemonic effects of liraglutide in a MWM task. Consequently, liraglutide treatment exhibited no effect on cerebral plaque load in two individual transgenic mouse models of low-grade (hAPP_Lon_/PS1_A246E_ mice) and high-grade (hAPP_Swe_/PS1_ΔE9_ mice) amyloidosis. This contrasts our recent finding that equivalent liraglutide dosing regimens are neuroprotective in non-transgenic SAMP8 mice [34] as well as in transgenic hTauP301L mouse model of tauopathy [35]. As these two models of AD display neurological deficits and histopathological changes in the absence of cerebral β-amyloid plaque accumulation, this may suggest differential sensitivity to long-term liraglutide treatment in various mouse models mimicking distinct pathological hallmarks of AD.
